# Variation in rates of ICU readmissions and post-ICU in-hospital mortality and their association with ICU discharge practices

**DOI:** 10.1186/s12913-017-2234-z

**Published:** 2017-04-17

**Authors:** Nelleke van Sluisveld, Ferishta Bakhshi-Raiez, Nicolette de Keizer, Rebecca Holman, Gert Wester, Hub Wollersheim, Johannes G. van der Hoeven, Marieke Zegers

**Affiliations:** 1 Radboud University Medical Center, Radboud Institute for Health Sciences, IQ healthcare, P.O. box 9101, 6500 HB Nijmegen, the Netherlands; 2 Stichting Nationale Intensive Care Evaluatie (NICE), Amsterdam Medical Centre, P.O. box 22660, 1105 AZ Amsterdam, The Netherlands; 3 Department of Medical Informatics, Amsterdam Medical Centre, P.O. box 22660, 1105 AZ Amsterdam, The Netherlands; 4 Department of Intensive Care Medicine, Radboud University Medical Center, P.O. box 9101, 6500 HB Nijmegen, The Netherlands

**Keywords:** Intensive care, Critical care, Variation, Mortality, Patient readmission, Patient discharge

## Abstract

**Background:**

Variation in intensive care unit (ICU) readmissions and in-hospital mortality after ICU discharge may indicate potential for improvement and could be explained by ICU discharge practices. Our objective was threefold: (1) describe variation in rates of ICU readmissions within 48 h and post-ICU in-hospital mortality, (2) describe ICU discharge practices in Dutch hospitals, and (3) study the association between rates of ICU readmissions within 48 h and post-ICU in-hospital mortality and ICU discharge practices.

**Methods:**

We analysed data on 42,040 admissions to 82 (91.1%) Dutch ICUs in 2011 from the Dutch National Intensive Care Evaluation (NICE) registry to describe variation in standardized ICU readmission and post-ICU mortality rates using funnel-plots. We send a questionnaire to all Dutch ICUs. 75 ICUs responded and their questionnaire data could be linked to 38,498 admissions in the NICE registry. Generalized estimation equations analyses were used to study the association between ICU readmissions and post-ICU mortality rates and the identified discharge practices, i.e. (1) ICU discharge criteria; (2) bed managers; (3) early discharge planning; (4) step-down facilities; (5) medication reconciliation; (6) verbal and written handover; (7) monitoring of post-ICU patients; and (8) consulting ICU nurses. In all analyses, the outcomes were corrected for patient-related confounding factors.

**Results:**

The standardized rate of ICU readmissions varied between 0.14 and 2.67 and 20.8% of the hospitals fell outside the 95% control limits and 3.6% outside the 99.8% control limits. The standardized rate of post-ICU mortality varied between 0.07 and 2.07 and 17.1% of the hospitals fell outside the 95% control limits and 4.9% outside the 99.8% control limits. We could not demonstrate an association between the eight ICU discharge practices and rates of ICU readmissions or post-ICU in-hospital mortality. Implementing a higher number of ICU discharge practices was also not associated with better patient outcomes.

**Conclusions:**

We found both variation in patient outcomes and variation in ICU discharge practices between ICUs. However, we found no association between discharge practices and rates of ICU readmissions or post-ICU mortality. Further research is necessary to find factors, which may influence these patient outcomes, in order to improve quality of care.

**Electronic supplementary material:**

The online version of this article (doi:10.1186/s12913-017-2234-z) contains supplementary material, which is available to authorized users.

## Background

Intensive care unit (ICU) readmissions pose an important clinical problem because they are associated with patient harm, inefficiencies and higher costs [1–4]. Patients readmitted to the ICU experience more adverse events, with in-hospital mortality rates up to six times higher than non-readmitted patients [5]. Readmitted patients reduce ICU bed availability and it is possible that ICU facilities could be used more efficiently if ICU readmissions could be prevented [1–4].

Risk factors for ICU readmission and in-hospital mortality following ICU discharge include patient characteristics, such as age, co-morbidities and severity of illness [5, 6], and organisational factors, such as discharge time and the availability of step-down facilities [5–8]. A substantial amount of variation in patient outcomes between hospitals may be explained by the organisation of the ICU discharge process [9], which consists of four essential components: decision making, planning and preparation, patient transport and follow-up. The ICU discharge process is a complex process in which many healthcare professionals are involved [10]. Deficits in communication, coordination of care, and information exchange between ICU and general ward professionals [11–13] may increase the risk of a suboptimal handover, severe adverse events, ICU readmissions and mortality [14]. Patients discharged from the ICU are particularly vulnerable to poor handovers due to the complicated physiology [15] and the substantial decrease in monitoring when these patients are transferred from the ICU to a general ward [16, 17].

Several methods and instruments are available which aim to improve the quality of the discharge of ICU patients to general wards, such as a liaison nurse and handover forms [18]. Evidence of the effectiveness of these interventions, however, is limited [18, 19] and the actual use of ICU discharge practices vary between ICUs [20].

Variation in ICU readmissions and in-hospital mortality after ICU discharge between hospitals may indicate potential for improvement and be explained by the ICU discharge practices which have been implemented. Insight into associations between ICU discharge practices and patient outcomes can provide evidence for professionals on ways to improve their ICU discharge process, and possibly, reduce adverse patient outcomes.

The aims of this study were: (1) to describe variation in rates of ICU readmissions within 48 h and post-ICU in-hospital mortality in individual hospitals; (2) to describe current ICU discharge practices in Dutch hospitals; and (3) to study the association between ICU discharge practices and rates of ICU readmissions within 48 h and post-ICU in-hospital mortality. We hypothesized that the implementation of ICU discharge practices would be associated with lower rates of ICU readmissions and lower rates of post-ICU in-hospital mortality.

## Methods

The design of the study was pre-specified and published [21].

### Patient data and outcomes

The Dutch National Intensive Care Evaluation (NICE) registry collects demographic, physiological, clinical and organizational data from ICUs. To ensure that the data are of a high quality, ICU employees are trained in how to score patients, the data are checked before being read into the database, and data quality audits are carried out [22–25].

We used data from the NICE registry on ICU admissions, for reasons other than cardiac surgery, between 1st January and 31st December 2011. We did not examine admissions following cardiac surgery, because cardiac surgery is only performed in a small number of hospitals in the Netherlands and these patients have a low risk of ICU readmission or post-ICU in-hospital mortality [1, 26]. We excluded admissions, in which the patient died during the initial ICU admission or was discharged from the ICU and hospital simultaneously, because these patients were not at risk for ICU readmission or post-ICU in-hospital mortality. We also excluded admissions not fulfilling the Acute Physiology and Chronic Health Evaluation (APACHE) IV inclusion criteria [26] and with missing data on type of admission, reason for discharge, APACHE physiology score, APACHE reason for admission or discharge location (Additional file 1).

We defined an initial ICU admission as a patient’s first ICU admission within a single hospital stay and an ICU readmission as the first ICU readmission within 48 h of the initial ICU discharge, but within the same hospital stay. We choose a time frame of 48 h, as readmissions within this period have a stronger relationship with ICU interventions, such as mechanical ventilation, and discharge circumstances, than later readmissions [27]. We defined post-ICU in-hospital mortality as the death of the patient after the initial ICU admission ended, but before he or she was discharged from the hospital.

### ICU discharge practices

Members of an expert panel, consisting of one internal medicine consultant, two intensive care consultants and two researchers, selected eight ICU discharge practices described in scientific literature and clinical guidelines [18, 19, 28–36] to examine in this study. We present these eight practices in Table 1. They were the use of: (1) ICU discharge criteria [29, 30]; (2) a bed manager [31, 32]; (3) early discharge planning [33] (4) step-down facilities [28, 30]; (5) medication reconciliation [32, 34]; (6) verbal and written handover [28, 30, 32]; (7) monitoring of post-ICU patients [36]; and (8) consulting ICU nurses [35]. We extracted data on the use of step-down facilities from the NICE registry. We collected data on the use of the other seven ICU discharge practices using an online questionnaire (Additional file 2), sent to all Dutch ICUs in May 2012. We sent reminders after 9 days and after 3 weeks and contacted the non-responding ICUs by telephone a month after initially sending out the questionnaire. We transformed the data on the use of the eight discharge practices into dichotomous variables to indicate the presence or absence of a discharge practice on a specific ICU (Additional file 3). We summed the eight dichotomous variables into a combined practice score, representing the number of discharge practices incorporated into the discharge process in each ICU.

**Table 1:** ICU discharge practices

Discharge practice	Description
Discharge criteria	the usage of set criteria when making the decision to discharge a patient from the ICU
Bed manager	nurse or physician managing bed availability in ICU and step-down facilities
Early discharge planning^a^	starting with planning a discharge at least 24 h before the transfer of the patient to the ward
Step-down facilities	beds with less monitoring and a lower nurse-patient ratio than ICU beds, but more monitoring and a higher nurse-patient ratio then ward beds.
Medication reconciliation^a^	creating an actual medication overview of current medications, (temporarily stopped) home medication, and information about allergies. Home medication and allergy information is checked with the patient or relatives.
Verbal and written handover^b^	oral and written information transfer by nurses, and oral and written information transfer by physicians
Monitoring of post-ICU patients	patients discharged from the ICU are visited on the ward and evaluated by ICU personnel
Consulting ICU nurses	an ICU nurse is 24/7 available for questions and assistance on the ward

^a^we asked what percentage of patients received early discharge planning or medication reconciliation. If the median percentage or more percent of the patients received the interventions, the ICU was deemed to have implemented this practice

^b^the ICU was deemed to have implemented this practice if all four forms of communication at discharge were performed: oral nursing handover, written nursing handover, oral medical handover, and written medical handover

### Statistical analyses

We calculated the standardised readmission and post-ICU mortality rates for each hospital by dividing the observed number of readmissions or deaths by the expected number of readmissions or deaths. The expected number of readmissions or deaths was the sum of the predicted probabilities of readmission or death obtained from separate prediction models. Readmission rates were corrected for ICU level (in which level 1 are the least and level 3 the most advanced ICUs), age, cardiovascular insufficiency, cirrhosis, hematological malignancy, cardio vascular accident, medical or surgical admission type, planned admission, mechanical ventilation in the first 24 h of admission, chronic renal insufficiency, chronic dialysis, chronic obstructive pulmonary disease, respiratory insufficiency, neoplasm, immunological insufficiency, gastrointestinal bleeding, acute renal failure, confirmed infection, vasopressors, and logit transformed APACHE IV mortality probability [26]. Mortality rates were corrected for ICU level (in which level 1 are the least and level 3 the most advanced ICUs), age, cardiovascular insufficiency, cirrhosis, hematological malignancy, cardio vascular accident, medical or surgical admission type, planned admission, mechanical ventilation in the first 24 h of admission, chronic renal insufficiency, chronic dialysis, chronic obstructive pulmonary disease, respiratory insufficiency, neoplasm, immunological insufficiency, gastrointestinal bleeding, acute renal failure, confirmed infection, vasopressors, diabetes, cerebrovasculair accident, CPR, dysrhythmia, and logit transformed APACHE IV mortality probability [26]. We assessed the discrimination of the prediction models using the area under the receiver operating characteristic (ROC) curve [37] and the calibration using the Hosmer-Lemeshow goodness-of-fit statistic Ĉ with 10 groups [38]. We presented the standardized rates in funnel plots with 95 and 99.8% control limits. We obtained the control limits under the assumption that the natural logarithms of the standardized rates follow a normal distribution [39]. ICUs outside the control limits can be interpreted as deviating significantly from the national rates.

We analysed the univariate association between ICU readmission and post-ICU in-hospital mortality and the eight ICU discharge practices using generalized estimation equations with a logit link function and robust variance estimators [40], while correcting for patient factors. We applied the Bonferroni correction to correct for multiple testing [41], and hence viewed the association between a ICU discharge practice and ICU readmission or post-ICU in-hospital mortality if *p*-value < 0.0056 (0.05/9). We performed the statistical analyses using IBM SPSS Statistics and R 2.13.0.

## Results

**Fig. 1:**
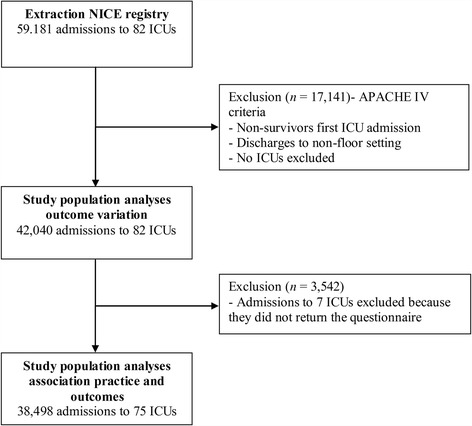
Flowchart of patients. NICE: national intensive care evaluation; ICU: intensive care unit; APACHE: acute physiology and chronic health evaluation

We extracted 59,181 first admissions to ICUs in 82 hospitals from the NICE registry (Fig. 1). We excluded 17,141 (Additional file 1) and included 42,040 admissions (71.0%) when calculating standardised readmission and post-ICU mortality rates. The ICUs were in six (7.3%) university hospitals, 29 (35.4%) teaching hospitals, and 47 (57.3%) general hospitals. We present the patient characteristics in Table 2.

**Table 2:** Patient characteristics

	(*n* = 42,040)
Median age in years (IQR)	65 (54 to 75)
Male (%)	23,832 (56.7)
Mechanical ventilation in the first 24 h of admission (%)	14,810 (35.2)
Vasoactive medication (%)	11,183 (26.6)
Planned admission (%)	12,918 (30.7)
Readmissions (%)	3463 (8.2)
Readmissions within 48 h of ICU discharge (%)	1216 (2.9)
Length of stay	
Median intensive care length of stay in days (IQR)	1.0 (0.80 to 2.9)
Median hospital length of stay in days (IQR)	11.0 (6.0 to 20.0)
Mortality	
Post-ICU in-hospital mortality rate (%)	2811 (6.7)
APACHE IV standardized mortality rate	
Median APACHE III score (IQR)	49 (49 to 68)
Mean APACHE IV probability (SD)	0.15 (0.19)
APACHE IV standardized mortality rate (95% CI)	0.78 (0.77 to 0.80)
Admission type:	
Medical/non-surgical (%)	18,324 (43.6)
Emergency surgery (%)	7139 (17.0)
Planned surgery (%)	16,577 (39.4)
Admission source:	
Operating theatre (%)	21,694 (51.6)
Emergency room (%)	8262 (19.7)
Ward (%)	9477 (22.5)
High or medium care (%)	159 (0.4)
Other hospital (%)	630 (1.5)
Other (%)	1818 (4.3)
Comorbidity on admission:	
Confirmed infection (%)	6300 (15.0)
Cardiopulmonary resuscitation (%)	1177 (2.8)
Dysrhythmia (%)	3136 (7.5)
Acute renal failure (%)	2658 (6.3)
Cardiovascular accident (%)	1513 (3.6)
Gastrointestinal bleeding (%)	977 (2.3)
Number of chronic comorbidities:	
None (%)	25,238 (60.0)
One (%)	11,538 (27.4)
Two (%)	4042 (9.6)
Three (%)	1029 (2.4)
More than three (%)	193 (0.6)
Patients discharged to:	
Ward (%)	39,493 (93.9)
Recovery or medium care (%)	1239 (3.1)
Coronary care unit or other intensive care unit (%)	1308 (3.0)

### Rates of ICU readmissions and post-ICU in-hospital mortality

**Fig. 2:**
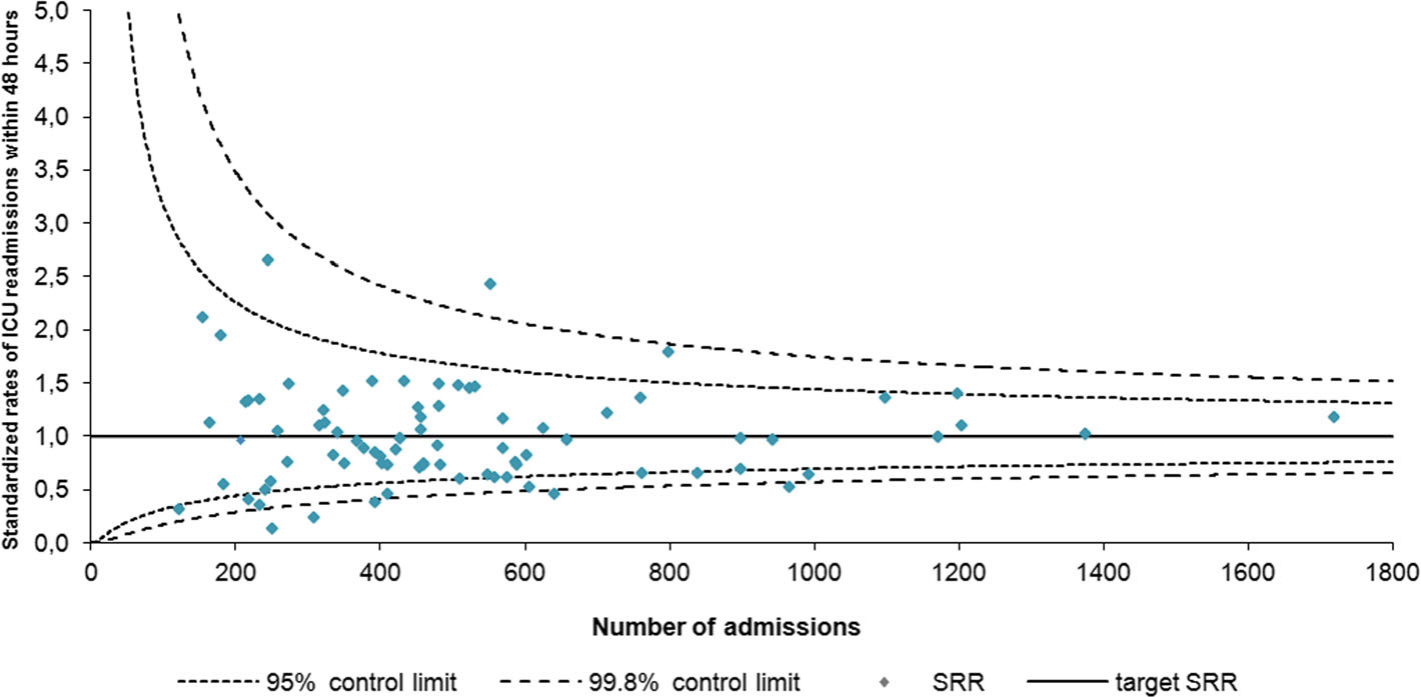
Standardized rates of ICU readmission within 48 h. Readmission rates were corrected for ICU level (in which level 1 are the least and level 3 the most advanced ICUs), age, cardiovascular insufficiency, cirrhosis, haematological malignancy, cardio vascular accident, medical or surgical admission type, planned admission, mechanical ventilation in the first 24 h of admission, chronic renal insufficiency, chronic dialysis, chronic obstructive pulmonary disease, respiratory insufficiency, neoplasm, immunological insufficiency, gastrointestinal bleeding, acute renal failure, confirmed infection, vasopressors, and logit transformed APACHE IV mortality probability [26]

We found a crude ICU readmission rate of 2.9% (1,216/42,040). The standardized rates varied between 0.14 and 2.7 with, by definition, an overall target rate of 1.00. In Fig. 2, we present a funnel plot of the standardized rates of ICU readmissions against the number of ICU admissions per ICU in 2011. In total, 65 (79.3%) ICUs fall within the 95% control limits, three (3.7%) above the upper, and 14 (17.1%) below the lower 95% control limits. One (1.2%) hospital falls above the upper and two (2.4%) hospitals fall below the lower 99.8% control limits. The calibration (Ĉ = 18.1, *p*-value = 0.0205) and discrimination (area under the ROC curve = 0.63) of the standardization model for ICU readmissions were poor.

**Fig. 3:**
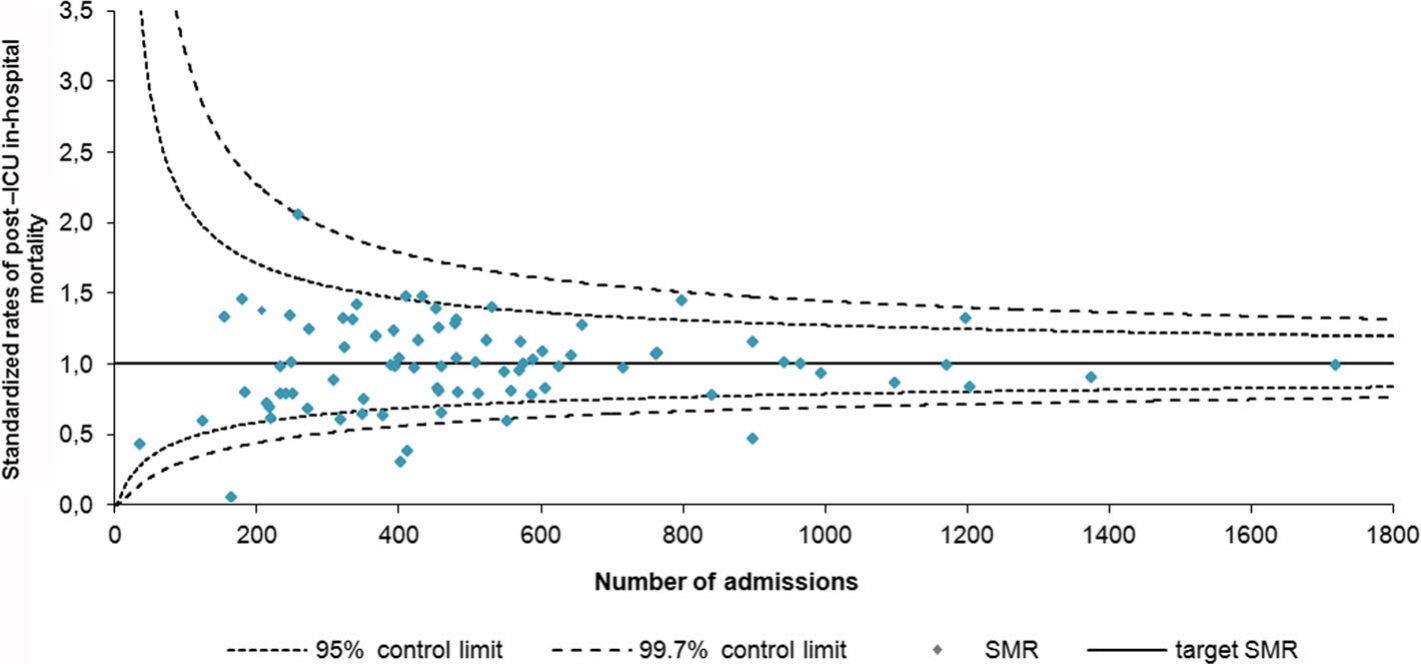
Standardized rates of post-ICU in-hospital mortality. Mortality rates were corrected for ICU level (in which level 1 are the least and level 3 the most advanced ICUs), age, cardiovascular insufficiency, cirrhosis, haematological malignancy, cardio vascular accident, medical or surgical admission type, planned admission, mechanical ventilation in the first 24 h of admission, chronic renal insufficiency, chronic dialysis, chronic obstructive pulmonary disease, respiratory insufficiency, neoplasm, immunological insufficiency, gastrointestinal bleeding, acute renal failure, confirmed infection, vasopressors, diabetes, cerebrovasculair accident, CPR, dysrhythmia, and logit transformed APACHE IV mortality probability [26]

We found a crude hospital mortality rate of 6.7% (2,811/42,040). The standardized rates ranged between 0.1 and 2.1. In Fig. 3, we present a funnel plot of these rates against the number of ICU admissions in 2011. Five hospitals (6.1%) have an adjusted post-ICU in-hospital mortality rate above the upper and nine (11.0%) below the lower 95% control limits. Four hospitals (4.9%) fall below the lower 99.8% control limits. Although the discrimination (area under the ROC curve = 0.82) of the standardization model for post-ICU in-hospital mortality was good, the calibration (Ĉ = 38.9, *p*-value < 0.0001) was poor.

### ICU discharge practices, ICU readmission and post-ICU in-hospital mortality

We had data from the NICE registry and a completed questionnaire for 75 ICUs (Additional file 4). To study the association between ICU discharge practices and ICU readmissions and post-ICU mortality, we excluded 3542 admissions to the non-participating ICUs. Hence, we used data on 38,498 admissions (65.1%) to 75 ICUs in the analyses on associations between ICU discharge practices and ICU readmission and post-ICU in-hospital mortality (Fig. 1). These ICUs were in six university hospitals (8.0%), 28 tertiary medical teaching hospitals (37.3%), and 41 general hospitals (54.7%). We present the results from the questionnaire in detail in Additional file 4.

Table 3 shows the percentages of ICUs, which had implemented each of the discharge practices, and the odds ratios of the univariate association between the implementation of each discharge practice and ICU readmission and post-ICU in-hospital mortality. Following the Bonferroni correction (*p* < (0,05/9) = *p* < 0.0056), none of the ICU discharge practices nor the total number of ICU discharge practices implemented by each ICU were associated with the standardized rates of readmission or mortality.

**Table 3:** Rates of individual practices and odds ratios of univariate association with patient outcomes

Individual practice rates in isolation					
Practices	*n* (%)	Case-mix adjusted^a^ readmission rate OR (95% CI)	*p*-value^b^	Case-mix adjusted^a^ post-ICU mortality rate OR (95% CI)	*p*-value^b^
Discharge criteria	53 (70.7)	0.95 (0.75–1.21)	0.6775	1.02 (0.83–1.24)	0.8541
Bed manager	71 (94.7)	1.08 (0.80–1.46)	0.6164	0.93 (0.52–1.68)	0.8128
Early discharge planning	40 (53.3)	1.04 (0.84–1.28)	0.7011	1.03 (0.89–1.20)	0.6667
Medication reconciliation	39 (52.0)	0.95 (0.78–1.17)	0.6587	1.00 (0.86–1.16)	0.9722
Communication at handover	49 (65.3)	0.90 (0.73–1.11)	0.9912	1.08 (0.92–1.28)	0.9442
Step-down facilities	21 (28.0)	1.21 (0.98–1.50)	0.0823	1.16 (1.01–1.34)	0.0423
Monitoring of post-ICU patients	49 (65.3)	1.02 (0.81–1.27)	0.8822	0.91 (0.78–1.07)	0.2654
Consulting ICU nurse	70 (93.3)	0.87 (0.64–1.19)	0.3948	0.90 (0.67–1.23)	0.5120
Combined practices score (median (IQR))	6 (5–7)	1.00 (0.93–1.10)	0.994	1.02 (0.95–1.08)	0.59548
Number of practices incorporated					
1	1 (1.3)				
2	3 (4.0)				
3	7 (9.3)				
4	10 (13.3)				
5	22 (29.3)				
6	18 (24.0)				
7	11 (14.7)				
8	3 (4.0)				

^a^Patient-related confounding factors for which is corrected are age, admission type (medical or surgical), planned admission, mechanical ventilation in the first 24 h, logit transformed APACHE IV mortality probability

^b^Significant odds ratio after Bonferroni correction (*p* < 0.0056 (= *p* < 0.05/9))

## Discussion

The objective of our study was to describe variation in ICU readmissions within 48 h and post-ICU in-hospital mortality and to study the association of these patient outcomes with the implementation of ICU discharge practices. Using funnel plots, we found that 20.8% of the ICUs fell outside the 95% control limits and 3.6% outside the 99.8% control limits with respect to ICU readmission and 17.1 and 4.9% with respect to post-ICU in-hospital mortality. The substantial proportion of ICUs with standardized readmission or mortality rates falling outside the control limits, suggests that there is more variation between hospitals on these patient outcomes than would be expected and that there is room for quality improvement. The ICUs with lower readmission or mortality can be viewed as best practices and ICUs with higher readmission or mortality could benefit from their views and experiences. The extent of this variation is consistent with that reported in studies on ICU length of stay and mortality [42, 43]. To study and to possibly explain the found variation, we subsequently studied the implementation of ICU discharge practices and their association with the occurrence of IC readmissions and post-ICU in hospital mortality rate.

In this study, we also found that ICU discharge practices vary. We had hypothesized that such variation could indicate that the ICU discharge process could be optimized and, hence, potentially improve patient outcomes and reduce healthcare costs [44, 45]. However, we were unable to demonstrate an association between ICU discharge practices and rates of ICU readmissions or post-ICU in-hospital mortality. In addition, implementing a higher number of ICU discharge practices was not associated with better patient outcomes. Results of previous studies reporting about the association between the use of patient safety and quality improvement practices, and patient safety outcomes are diffuse. Some studies showed that compliance to discharge practices was associated with lower hospital complications and mortality rates [46, 47], while others showed no association [48].

An important strength of this study is our large dataset covering more than 90% of all Dutch ICUs. In addition, we included the APACHE IV mortality probability in our case-mix correction models. Currently, the APACHE IV is the best performing model for case-mix correction for in-hospital mortality following ICU admission in the Netherlands [25, 26].

Our study has some limitations. We strived to minimize the effects of case-mix differences between ICUs by presenting case-mix adjusted standardized rates for quantifying variation in patient outcomes. However, our case-mix correction models have not been externally validated and, in our dataset, the calibration of the models for ICU readmission and post-ICU mortality and the discrimination of the model for ICU readmission were poor. This means that these models may not adequately correct for case-mix differences between hospitals, potentially resulting in more hospitals than expected falling outside the control limits [49]. In addition, the variation between the rates of ICU readmission and post-ICU in-hospital mortality may still result from chance [50], overdispersion [39, 51], an incorrect method for determining the control limits [52], or registration problems within the hospitals.

The number of admissions included in our analyses for some ICUs was very low. Therefore, even when using funnel plots, there is a low probability of detecting that these ICUs are performing differently from national rates [53].

We found no significant association between ICU discharge practices and patient outcomes which may be due to several limitations of our study. First, the power to detect a reduction in post-ICU mortality and ICU readmission rate was limited because we measured each of the discharge practices at hospital level and, although the response rate to the questionnaire was 91.5%, the number of ICUs was limited. Furthermore, some practices were present in almost every ICU. Second, the use of discharge practices were measured using a self-reported questionnaire, which may be susceptible to bias. Overestimation of own practices and socially desirable answers could have influenced our findings. However, Scholle and colleagues found only minor overestimation in their study and concluded that self-assessment could be useful for quality improvement purposes [54]. Third, in our regression models we used patient data, such as severity of illness and the APACHE IV reasons for ICU admission, measured at the time of ICU admission. Ideally, data representing the patient’s condition at the time of ICU discharge would be used. However, these data are not available in the NICE registry. Fourth, we had no data on whether patients were discharged from the ICU for palliative care on the ward. This could have led to an overestimation of the mortality rates.

Clinical handover has been identified as a key process in improving quality of care and patient safety and reducing adverse patient outcomes [55, 56]. Quantification of variation is a tool for uncovering suboptimal quality of care and may identify potential for improvement [57–59]. We found both variation in patient outcomes and in discharge practices and reasoned that this indicates potential for improving patient outcomes and subsequently, reducing healthcare costs [44, 45]. However, we were not able to identify a relation between ICU discharge practices and patient outcomes. Further research is necessary to find factors, which may influence these patient outcomes, in order to improve quality of care. For example organisational factors, such as staffing and experience and skills of (ICU) personnel. Unfortunately, we were not able to include them in our research due to the lack of data of these factors. Exploratory research into the differences between the hospitals falling above the upper and below the lower control limits in our funnel plots may give insight into factors influencing quality of care.

## Conclusion

Causes of ICU readmissions and post-ICU in-hospital mortality are likely to vary between hospitals. Although interventions to reduce the rates of these events have been described in the literature, our study shows that none of them are associated with better outcomes in the Netherlands. Examining individual ICU readmissions or post-ICU in-hospital mortalities locally may provide ICUs insight into potential areas for improvement in their own ICU discharge process.

## Additional files

Additional file 1: Number of admissions excluded per exclusion criterion. (PDF 83 kb)

Additional file 2: Questionnaire ‘ICU discharge practice’ translated from Dutch to English. (PDF 112 kb)

Additional file 3: Dichotomising Questionnaire variables. (PDF 80 kb)

Additional file 4: Results questionnaire. (PDF 99 kb)

Additional file 5: Database Questionnaire (XLSX 37 kb)

## Abbreviations

APACHE: Acute physiology and chronic health evaluation
ICU: Intensive care unit
NICE: National intensive care evaluation
ROC: Receiver operating characteristic
