# Supplementary material for: Variation in rates of ICU readmissions and post-ICU in-hospital mortality and their association with ICU discharge practices
Source: BMC Health Serv Res. 2017 Apr 17;17:281. doi: 10.1186/s12913-017-2234-z (PMC5393034; doi:10.1186/s12913-017-2234-z)
Supplement: Supplementary file 1 — Number of admissions excluded per exclusion criterion. (PDF 83 kb) [file 12913_2017_2234_MOESM1_ESM.pdf]

Additional file 1. Number of admissions excluded per exclusion criterion.

	n
Total excluded from regression analyses	17,141[a]
ICU non-survivors during first intensive care unit admission	6213
ICU survivors discharged to a non-floor location	6151
**APACHE IV exclusion criteria**	
Age less than 16 years	341
Length of stay less than 4 hours	2683
Length of stay greater than 365 days	5
Died before admission	69
Patients with substantial burns	75
Patients with transplants (except renal and hepatic)	123
Admission from another intensive care unit	2807
Missing admission type	1254
Missing hospital discharge type	270
Missing APACHE III score	69
Missing APACHE IV reason for admission	1104
Missing discharge location	234

[a] The individual numbers in this table do not sum to the total, because some admissions are excluded for multiple reasons.
