# Supplementary material for: Variation in rates of ICU readmissions and post-ICU in-hospital mortality and their association with ICU discharge practices
Source: BMC Health Serv Res. 2017 Apr 17;17:281. doi: 10.1186/s12913-017-2234-z (PMC5393034; doi:10.1186/s12913-017-2234-z)
Supplement: Supplementary file 2 — Questionnaire ‘ICU discharge practice’ translated from Dutch to English. (PDF 112 kb) [file 12913_2017_2234_MOESM2_ESM.pdf]

Additional file 2. Questionnaire 'ICU discharge practice' translated from Dutch to English.

Demographic question

Question 1.

What is your function title?

- Head of ICU / medical manager
- Intensivist
- Fellow
- Nurse
- Other, namely ...

Discharge policy

Question 2.

Is policy concerning the discharge of ICU patients written down?

- There is policy, approved by the medical staff
- There is policy, approved by the medical staff and approved by the hospital board
- There is policy, approved by the medical staff, approved by the hospital board and periodically tested and reported to the medical staff and hospital board
- Policy is being developed
- There is no policy written down

Question 3.

Is the NVIC guideline 'criteria voor opname en ontslag van de intensive care-afdelingen in Nederland' translated to a protocol?

- Yes
- No
- Being developed

Question 4. (only if Question 3. was answered with 'yes')

Is this protocol available on the ICU? (multiple answers are possible)

- Yes, on paper
- Yes, electronically
- Yes, with decision support
- No

Question 5.

Are there set discharge criteria?

- Yes
- No
- Being developed

Question 6.
Who takes the decision to discharge a patient from the ICU? (multiple answers possible)

- o Intensivist
- o Fellow
- o Resident
- o Nurse
- o Other, namely ...

Question 7.
Is the responsibility for the discharge decision written down?

- o Yes
- o No

*Question 8.**
On which items is a discharge decision or the postponement of discharge based? (multiple answers possible)

o Set discharge criteria	in ... % of the patients
o Knowledge / view / experience of care professional	in ... % of the patients
o Logistic reasons (i.e., strain on ICU or ward beds)	in ... % of the patients
o Arguments based on nursing (i.e., nursing load)	in ... % of the patients
o Other, namely ...	in ... % of the patients

Preparation for discharge

*Question 9.**
Is there a person who keeps track of the number of available beds on the ICU as well as on the step-down facilities?

- o Yes, an intensivist
- o Yes, a fellow / resident
- o Yes, a nurse
- o No

*Question 10.**
Can you give an estimation of the percentage of patients with a ICU length of stay of more than 24 hours, which received 'early discharge planning'?
...%
(Definition early discharge planning: the preparation for patient discharge, such as coordination with the receiving ward about discharge time, starts at least 24 hours before actual transport of the patient.)

Handover

Question 11.
Is the medical and nursing handover separated?

- o Yes
- o No

*Question 12.**
At ICU discharge: (multiple answers possible)
- o a written or electronic nursing discharge form goes along with the patient
- o verbal handover between nurses takes place
- o a letter with medical data, medication and treatment advice is send to the receiving ward directly
- o verbal handover between physicians takes place
- o Other, namely …

Question 13.
A structured handover consists of: (multiple answers possible)
- o a summary of the ICU admission, including diagnosis and treatment
- o monitoring plan and planning of medical tests
- o treatment plan, including medication, treatments, diets, infection status and treatment limitations
- o list of medications, including stopped medications and (changes in) medications used at home
- o allergy information
- o information about revalidation needs (i.e., physiotherapy, speech therapy)
- o specific communication and language needs
- o Other, namely …

Handover of medication information
*Question 14.**
Can you give an estimation of the percentage of patients which received 'medication reconciliation'?
…%
(Definition medication reconciliation: creating a recent overview of current medications, (temporarily stopped) home medications and information about possible allergies. Home medication and allergies are verified with the patient or his or her relatives.)

After care
*Question 15.**
Is general ward staff able to ask 24/7 for help or advice from a consulting ICU nurse about post-ICU patients?
- o Yes
- o No

Question 16.
Is help by the ICU arranged in certain nursing activities?
- o Yes, on a regular basis
- o Yes, incidentally
- o No

*Question 17.**

Are post-ICU patients monitored on the wards? (multiple answers possible)

- o Yes, by a (consulting) ICU nurse in … % of the patients
- o Yes, by an intensivist in … % of the patients
- o Other, namely … in … % of the patients
- o Are not visited

Question 18.

Does your hospital have a medical emergency team (MET)?

- o Yes
- o No
- o Being developed

Concluding questions

Question 19.

Does your hospital use other practices than mentioned in this questionnaire to organize patient discharge from ICU to general ward?

(open question)

Question 20.

Are there any barriers at patient discharge from ICU to general ward, and if yes, can you describe them?

(open question)

Question 21.

Is there anything you like mention what was not yet mentioned in the questionnaire?

(open question)

Question 22.

Do you consent to linking the data from this questionnaire with data from the NICE registry? The linking will be done by staff of NICE and the results will be reported anonymized and aggregated.

- o Yes
- o No

* Questions used to analyze the association between ICU discharge practices and ICU readmission and post-ICU in-hospital mortality. Variables related to the use of a set discharge policy and the use of the Dutch guideline were excluded from further analysis because they did not comprise of practices which in themselves may improve quality. Policy and guidelines are used as methods to implement practices which aim to improve the quality of the discharge process. The variables related to the medical emergency team were also excluded from further analysis. After deliberation with experts of intensive care it was decided that this practice was unlikely to have an impact on the ICU discharge process, and thus was outside the scope of the study.
