# Supplementary material for: Variation in rates of ICU readmissions and post-ICU in-hospital mortality and their association with ICU discharge practices
Source: BMC Health Serv Res. 2017 Apr 17;17:281. doi: 10.1186/s12913-017-2234-z (PMC5393034; doi:10.1186/s12913-017-2234-z)
Supplement: Supplementary file 3 — Dichotomising Questionnaire variables. (PDF 80 kb) [file 12913_2017_2234_MOESM3_ESM.pdf]

Additional file 3. Dichotomising Questionnaire variables.

Question	Answers	Dichotomous variables
On which items is a discharge decision or the postponement of discharge based?	Set discharge criteria yes/no<br>Knowledge/view/experience care professional yes/no<br>Logistic reasons yes/no<br>Arguments based on nursing yes/no<br>Other yes/no	Set discharge criteria yes (1)<br>Set discharge criteria no (0)
Is there a person who keeps track of the number of available beds on the ICU as well as on the step-down units?	Yes, an intensivist<br>Yes, a fellow/resident<br>Yes, a nurse<br>No	Yes, an intensivist (1)<br>Yes, a fellow/resident (1)<br>Yes, a nurse (1)<br>No (0)
Can you give an estimation of the percentage of patients with a ICU length of stay of more than 24 hours, which received 'early discharge planning'?	[1-100]%	Median = 20 →<br>0-19% (0)<br>20-100% (1)
At ICU discharge:	A written or electronic nursing discharge form goes along with the patient yes/no<br>Verbal handover between nurses takes place yes/no<br>A letter with medical data, medication and treatment advice is send to the receiving ward directly yes/no<br>Verbal handover between physicians takes place yes/no<br>Other yes/no	If 'a written or electronic nursing discharge form goes along with the patient' is yes AND 'verbal handover between nurses takes place' is yes AND 'a letter with medical data, medication and treatment advice is send to the receiving ward directly' is yes AND 'verbal handover between physicians takes place' is yes (1)<br>All other options (0)
Can you give an estimation of the percentage of patients which received 'medication reconciliation'?	[1-100]%	Median = 95 →<br>0-94% (0)<br>95-100% (1)
Is general ward staff able to ask 24/7 for help or advice from a consulting ICU nurse about post-ICU patients?	Yes<br>No	Yes (1)<br>No (0)
Are post-ICU patients monitored on the wards?	Yes, by a (consulting) ICU nurse<br>Yes, by an intensivist<br>Other<br>No	Yes, by a (consulting) ICU nurse (1)<br>Yes, by an intensivist (1)<br>Other (1)<br>No (0)
