# Supplementary material for: Variation in rates of ICU readmissions and post-ICU in-hospital mortality and their association with ICU discharge practices
Source: BMC Health Serv Res. 2017 Apr 17;17:281. doi: 10.1186/s12913-017-2234-z (PMC5393034; doi:10.1186/s12913-017-2234-z)
Supplement: Supplementary file 4 — Results questionnaire. (PDF 99 kb) [file 12913_2017_2234_MOESM4_ESM.pdf]

Additional file 4. Results questionnaire*

No.	Question		*n* (%)
**1.**	**Function title**		
		Head of ICU / medical manager	27 (36.0)
		Intensivist	42 (56.0)
		Fellow	0 (0.0)
		Nurse	3 (4.0)
		Other	3 (4.0)
**2.**	**Discharge policy**		
		Yes, approved by medical staff	32 (42.7)
		Yes, approved by medical staff and approved by hospital board	29 (38.7)
		Yes, approved by medical staff, approved by	
		hospital board, and periodically tested and	8 (10.7)
		reported to medical staff and hospital board	1 (1.3)
		Being developed	5 (6.7)
		No	
**3.**	**Protocol NVIC guideline**		
		Yes	68 (90.7)
		No	4 (5.3)
		Being developed	3 (4.0)
**4.**	**Protocol available (multiple answers possible)**		(n = 68)
		Yes, on paper	21 (28.0)
		Yes, electronically	63 (84.0)
		Yes, with decision support	0 (0.0)
		No	0 (0.0)
**5.**	**Set discharge criteria**		(n = 74)
		Yes	66 (88.0)
		No	6 (8.0)
		Being developed	2 (2.7)
**6.**	**Discharge decision (multiple answers possible)**		75 (100.0)
		Intensivist	4 (5.3)
		Fellow	2 (2.7)
		Resident	3 (4.0)
		Nurse	2 (2.7)
		Other	
**7.**	**Responsibility discharge decision**		
		Yes	69 (92.0)
		No	6 (8.0)
**8.**	**a. Basis on which discharge decision is taken (multiple answers possible)**		
		Discharge criteria	53 (70.7)
		Knowledge / insight / experience	70 (93.3)
		Logistic reasons	56 (74.7)
		Arguments based on nursing	63 (84.0)
		Other	14 (18.7)
	**b. Percentage of patients discharged on specific basis [*n* (mean percentage/median percentage)]**		48
		Discharge criteria	(66.2/80)
		Knowledge / insight / experience	64
		Logistic reasons	(63.5/80)

	Arguments based on nursing	51 (8.2/5)
	Other	55 (10.3/5)
		11 (13.2/1)
**9.**	**Bed manager**	
	Yes, intensivist	52 (69.3)
	Yes, fellow / resident	4 (5.3)
	Yes, nurse	38 (50.7)
	No	4 (5.3)
**10.**	**Percentage of patients with early discharge planning [*n* (mean percentage/median percentage)]**	72 (27.7/20)
**11.**	**Separated medical and nursing handover**	
	Yes	72 (96.0)
	No	3 (4.0)
**12.**	**Methods of communication (multiple answers possible)**	74 (98.7)
	Written or electronic nursing discharge form	73 (97.3)
	Verbal nursing handover	62 (82.7)
	Medical discharge summary	54 (72.0)
	Verbal medical handover	8 (10.7)
	Other	
**13.**	**Content structured handover (multiple answers possible)**	74 (98.7)
	Summary ICU admission	39 (52.0)
	Monitoring plan en planning of tests	70 (93.3)
	Treatment plan	72 (96.0)
	List of medications	66 (88.0)
	Allergy information	58 (77.3)
	Revalidation information	31 (41.3)
	Communication needs	12 (16.0)
	Other	
**14.**	**Percentage of patients with medication reconciliation [*n* (mean percentage/median percentage)]**	73 (75.0/95)
**15.**	**Consulting ICU nurse**	
	Yes	70 (93.3)
	No	5 (6.7)
**16.**	**Help with nursing activities**	
	Yes, regularly	53 (70.7)
	Yes, incidentally	22 (29.3)
	No	0 (0.0)
**17.**	**a. Monitoring post-ICU patients**	
	Yes, Consulting ICU nurse	45 (60.0)
	Yes, intensivist	11 (14.7)
	Yes, other	15 (20.0)
	No	26 (34.7)
	**b. Percentage of patients monitored by specific person [*n* (mean percentage/median percentage)]**	40
	Yes, Consulting ICU nurse	(49.9/50)
	Yes, intensivist	10
	Yes, other	(16.0/10)
		12
		(66.7/80)

**18.**	**Medical emergency team**	
	Yes	70 (93.3)
	No	0 (0.0)
	Being developed	5 (6.7)

*Not all questions were included in the analyses
